# Editorial: Biocompatible hydrogels: properties, synthesis and applications in biomedicine

**DOI:** 10.3389/fchem.2024.1500836

**Published:** 2024-10-09

**Authors:** Lei Nie, Carolina Muñoz-Camargo, Sayan Ganguly, Lahoucine Bahsis, Juan C. Cruz, Reza Mohammadinejad, Aldo Nicosia, Luis H. Reyes, Xing Wang

**Affiliations:** ^1^ College of Life Sciences, Xinyang Normal University, Xinyang, China; ^2^ Department of Biomedical Engineering, Faculty of Engineering, University of Los Andes, Bogotá, Colombia; ^3^ Department of Chemistry, University of Waterloo, Waterloo, ON, Canada; ^4^ Laboratoire de Chimie Analytique et Moléculaire, LCAM, Faculté Poly Disciplinaire de Safi, Université Cadi Ayyad, Safi, Morocco; ^5^ Kerman University of Medical Sciences, Kerman, Iran; ^6^ Department of “Scienze e Tecnologie Biologiche, Chimiche e Farmaceutiche” (STEBICEF), Institute for Biomedical Research and Innovation—National Research Council (IRIB-CNR), University of Palermo, Palermo, Italy; ^7^ Department of Chemical and Food Engineering, University of Los Andes, Bogotá, Colombia; ^8^ Institute of Chemistry, Chinese Academy of Sciences (CAS), Beijing, China

**Keywords:** hydrogels, biocompatible hydrogels, hydrogels preparation, hydrogels applications hydrogels engineering, hydrogel rational design, natural polymers, synthetic polymers, biomedical applications

It gives us great pleasure to prepare this invited editorial for the Research Topic in Frontiers in Chemistry titled “*Biocompatible Hydrogels: Properties, Synthesis and Applications in Biomedicine*.” Due to the favourable mechanical properties, biocompatibility and biodegradability features, the hydrogels with three-dimensional (3D) networks have displayed great potentials in biomedical applications, including drug delivery and tissue engineering. In recent years, several strategies and techniques on biocompatible polymer constructions and hydrogel preparation have been developed, guaranteeing the biocompatible hydrogels in several biomedical applications, including wound healing, cancer therapy, etc. In this Research Topic, a number of interesting studies were written by 42 authors, related to biocompatible hydrogels and polymeric nano-materials with functional characteristics in tissue engineering and biomedicine applications. Niclosamide has been widely used in treating cancer, viral infections, and metabolic disorders due to its ability to regulate signalling pathways and biological processes. To improve the efficacy of niclosamide, Tai et al. have fabricated niclosamide conjugated microspheres based on poly(lactic-*co*-glycolic acid) (PLGA) and hyaluronic acid (HA) via microfluidic approach. The obtained microspheres with a drug-loading efficiency of 8.70% displayed a sustained release behavior at pH 7.4 and 5.0, showing a quasi-first-order release kinetic. Furthermore, the niclosamide conjugated microspheres could increase the cellular uptake using Caco-2 cells and the inhibitory effect on Caco-2 cells, showing potential in targeted drug delivery in the intestine. In recent years, cancer immunotherapy using the hydrogels strategies has made significant progress. Liu et al. have summarized the objective of hydrogels in cancer immunotherapy in the last 10 years using bibliometric analysis based on the Web of Science Core Collection database. They have revealed that the primary research hotspots mainly include cancer immunotherapy, drug delivery, immunogenic cell death, tumor microenvironment, injectable hydrogels, and immune checkpoint blockade. Especially adoptive T-cell therapy, cell capture, adaptive cell therapy, photodynamic therapy, and sustained release have become research hotspots in the last 3 years. In the cancer therapy field, boron neutron capture therapy has been demonstrated as a highly targeted and effective treatment in curing various types of cancers with less damage to healthy cells. Cao et al. have reported a polymer Plnd containing a polyindole structure for labelling the modified *o*-carborane, and the problem of poor bioavailability of carborane was solved. They confirmed that the compound A-Plnd-C synthesized using methyl methacrylate (MMA), butyl methacrylate (BMA), diethylaminomethyl methacrylate (DEAMEA), and Plnd-C, had the best UV absorption and fluorescence intensity. The synthesized A-Plnd-C could enter HaLa and HCT-116 cells, and also exhibited pronounced inhibitory effects on HeLa, PC-3, and L02 cells.

Functional hydrogels have been commonly used in biomedical applications, including regenerative repair and drug delivery. Han et al. have developed a multifunctional hydrogel based on phenylboronic acid modified chitosan and dopamine modified hyaluronic acid via the boric acid ester bonds crosslinks. The obtained hydrogels with porous microstructure displayed elastic solid behavior, shear-thinning, and adhesion characteristics. Antibacterial tests and *in vitro* investigations also confirm the fabricated hydrogels possess efficient antibacterial activity and excellent cytocompatibility, displaying their potential as wound dressings within the realm of wound healing. In a review paper, Zhong et al. summarized the construction methods and biomedical applications of polyvinyl alcohol (PVA)-based hydrogels due to favourable properties of inexpensive and stable PVA, including excellent mechanical strength and biocompatibility. The most attractive preparation methods based on non-covalent interactions and chemical cross-linking for PVA-based hydrogels were summarized. The main applications for articular cartilage restoration, electronic skin, and wound dressing in recent years are discussed in this paper.

In another research, Azeroual et al. have developed a low-cost, efficient, and reusable cryogel beads adsorbent based on sodium alginate and titanium dioxide nanowire doped with zirconium for moving dyes from the wastewater. This obtained adsorbent exhibits the impressive adsorption capacities of methylene blue and safranin, displaying a pseudo-second-order adsorption kinetic model. The applications of biocompatible hydrogels have been broadened. Via the rational design in hydrogels constructions, the biocompatible hydrogels have represented great potential in tissue engineering and biomedicine. Therefore, the main aim of this Research Topic is to underpin the importance of biocompatible hydrogels in biomedical applications. For instance, Han et al. have shown a multifunctional composite hydrogel as wound dressing, Tai et al. have introduced the drug conjugated microspheres, and Cao et al. have synthesized a novel polymer for drug delivery. We believe that the encouraging approaches could advance the biocompatible hydrogels in tissue engineering and drug delivery, as well as for future clinical applications. Once again, we sincerely hope the researchers in related areas will enjoy reading all the papers in this special edition.

